# 114 Prime Time of Life: A multimodal physical activity and health education programme for middle-aged and older adults

**DOI:** 10.1093/eurpub/ckae114.181

**Published:** 2024-09-26

**Authors:** Louise Burke, Caroline Myers

**Affiliations:** Sport Ireland, Dublin, Ireland; Sport Ireland, Dublin, Ireland

## Abstract

Prime Time of Life is inclusive of people living with clinical conditions and differing fitness and functional abilities. Health screening was conducted preprogramme, and specific clinical exercise advice and support was provided to participants by Dr Diane Cooper, Clinical Exercise Physiologist. Participants completed two 60-minute multimodal (cardiovascular fitness, strength, balance, flexibility) exercise classes per week and were also encouraged to complete additional classes in their purpose built on-demand exercise library. In all 60-minute exercise classes the exercise instructor demonstrated three different options for each exercise to cater for participants who were working at beginner, intermediate and advanced levels of fitness. Participants appreciated the adaptations and progressions that were available for all exercises as it enabled them to work to their ownability and progress and monitor progress throughout the programme. Each week a 20-minute health education workshop relevant to participants was delivered online via Zoom. The workshop was recorded, edited, and uploaded to YouTube to view on-demand. The link for each video was shared with participants weekly via email to support on-demand learning in addition to any other relevant supporting material such as a training diary.

The Prime Time of Life programme increased participants upper and lower body strength, aerobic fitness and perceptions of physical and mental health (see chapter 3). This was in addition to increased confidence and feelings of positivity around the participants’ own management of healthy functional ageing (see chapter 3). Participants commented that they really enjoyed the Prime Time of Life programme. It increased their awareness on the importance of multimodal exercise for healthy ageing and that they felt empowered to continue training and improve their health in the long term.

Following on from this successful programme a full train the trainer programme has been developed and piloted with 9 Local Sports Partnerships, 20 participants with further plans for upscaling.

